# Cross-cultural adaptation of the drug-taking confidence questionnaire drug version for use in Brazil

**DOI:** 10.1186/s12874-016-0153-z

**Published:** 2016-05-18

**Authors:** Selene Cordeiro Vasconcelos, Everton Botelho Sougey, Iracema da Silva Frazão, Nigel Ernest Turner, Vânia Pinheiro Ramos, Murilo Duarte da Costa Lima

**Affiliations:** Federal University of Paraíba-UFPB, João Pessoa, Brazil; Federal University of Pernambuco-UFPE, Recife, Brazil; University of Toronto and Independent Scientist, Center for Addiction and Mental Health, Toronto, Canada

**Keywords:** Self-efficacy, Nursing, Drug users, Mental health, Addiction, Cross-cultural adaptation, Instrument translation, Validation studies

## Abstract

**Background:**

The Drug-Taking Confidence Questionnaire evaluates a drug user’s confidence in his or her ability to resist the urge to consume psychoactive substances in high-risk situations. This study’s objective was to develop a cross-cultural adaptation of the eight-item version of the Drug-Taking Confidence Questionnaire (DTCQ-8) for all drugs except alcohol and to verify its content validity and reliability in a pre-test stage.

**Methods:**

The following steps were taken: (1) implementation of the translation protocol and transcultural adaptation, (2) validation of the adapted content, and (3) assessment of reliability. Nine experts participated in the process of adaptation, and the trial’s sample comprised 40 drug users in treatment at a Psychosocial Care Center for Alcohol and Other Drugs (CAPSad).

**Results:**

The average indices of semantic agreement (0.989; 0.989; 1.00), idiomatic (0.967), experiential (0.956), conceptual (0.978) and content validity with respect to language clarity (0.972), practice relevance (0.958), theoretical relevance (0.958) and theoretical dimension (1.00) showed that the adaption was successful. The mean total score of the DTCQ-8 version for other drugs was 477.00 + 234.27-SD, and 57.5 % of the users were classified as having moderate self-efficacy to resist the urge to use drugs in high-risk situations. The Cronbach’s alpha coefficient was 0.889 for the complete instrument and 0.863–0.890 between items.

**Conclusions:**

The DTCQ-8 version for other drugs proved to be easy to use and understand, and its process of adaptation was satisfactory for use in the Brazilian context. In this sample, the questionnaire was adequate to measure users’ self-efficacy to resist the urge to consume these substances in high-risk situations.

## Background

Abuse and drug addiction are major public health problems worldwide [1]. These problems are characterized by users’ drug-seeking behavior, loss of pleasure in daily activities, and deterioration of social, work and family relationships [2, 3]. The Brazilian government recognizes these issues and has been investing in programs to treat drug users as well as addressing the implications of this use [4].

Consumption of stimulating drugs, such as cocaine, smoked or snorted, is increasing in Brazil, whereas in other emerging countries it has been declining [5]. Approximately 5 million Brazilian adults have snorted cocaine at least once in their lifetime, and approximately 2 million said they had used it in the previous 12 months. In addition, 1.7 million Brazilian adults have used smoked cocaine (crack) at least once in their lifetime, and 800,000 used it in the previous 12 months [6]. Crack use has been associated with high mortality among users [7].

The prevalence of smokers in Brazil is 15.0 % (21.9 million people); commercial cigarettes are the most commonly used tobacco product. Men constitute a higher percentage of users (19.2 %) than women (11.2 %), and the highest percentage of users (19.4 %) are 40–59 years of age [8].

Among the many harmful effects of drug abuse, the most common are cognitive and behavioral changes [9–11] and impaired self-efficacy, which is the confidence in one’s ability to organize and execute behaviors necessary to manage life [12, 13]. Self-efficacy has been integrated into social cognitive theory [13], which is based on the agency for self-development, adaptation and change [14, 15].

Self-efficacy is the basis of various treatment strategies for drug abusers [16–18]. Validated instruments designed to measure a particular aspect of drug use to guide interventions in health care can contribute to the development of more appropriate strategies and alleviation of the losses due to the harmful use of drugs [19, 20].

Measurement of self-efficacy to resist the urge to consume psychoactive substances in high-risk situations has been related to outcomes and treatment prognosis for drug users as well as the rates of recovery from the problems arising from such consumption [21–24]. Therefore, a valid and reliable instrument to measure this self-efficacy is an important tool for planning and evaluating the health interventions in that population [24].

To assess this self-efficacy—that is, to assess drug users’ trust in their ability to confront these situations—Annis and Martin (1985) [25] created the Inventory of Drug-Taking Situations. Later Sklar, Annis and Turner (1997) [26] developed the Drug-Taking Confidence Questionnaire. This version, which contained 50 items (DTCQ-50), was an explanatory guide [27], and its confirmed validity led to the release of the eight-item version (DTCQ-8) [28].

The DTCQ-8, which was validated only for heroin users, was translated into Spanish by Del Pozo [29] and German by Demmel [unavailable]. Despite the existence of a cross-cultural adaptation of the instrument, only the English-language version was used in other countries. Therefore, the present study’s objective was to conduct a cross-cultural adaptation and translation of the DTCQ-8 for other drugs except alcohol and to verify its face validity, content and reliability in the pre-test stage, which will support the clinical process of validation in the Brazilian context.

The adapted DTCQ-8 version for other drugs may help Brazilian researchers in choosing their research methods and, ultimately, assist in the identification, evaluation, and monitoring of, and intervention in, high-risk situations for substance users. The outcomes achieved by health professionals will benefit from research tools that are useful for clinical practice.

## Methods

### Participant recruitment and assessment

The sample was consecutive, intentional and non-probabilistic, with the following inclusion criteria: use of other drugs (other than alcohol), age 18–65 years, and undergoing treatment at CAPSad for at least a month. Exclusion criteria were cognitive limitations or medical and/or psychological states of health.

### Self-efficacy score

The DTCQ-8 total score was calculated by averaging each user’s scores for the eight items, and the self-efficacy level classification for each participant was based on the individual DTCQ-8 total score. Users were classified as having low self-efficacy if their individual DTCQ-8 total score was below 20 %, moderate self-efficacy if the score was 20–80 %, and high self-efficacy if the score was above 80 %.

### Cross-cultural adaptation of the DTCQ-8 drug version for use in Brazil

This was a methodological study of a cross-cultural adaptation of the DTCQ-8 for use in the Brazilian context. The objective was to achieve equivalence between the original version (developed in Canada) and the adapted version (for Brazil); it was essential for all items in the Brazilian version to maintain the validity of the instrument on a conceptual level [30, 31]. The process was performed in accordance with the five steps described by Beaton et al. (2007) [30], which in turn were derived from work by Guillemin et al. (1993) [32]. Beaton and collleagues’ procedure, described below, provides an important model for cross-cultural adaptation of health-status self-report questionnaires.

- Step 1: Initial translation (T1 and T2): the DTCQ-8 version for other drugs was translated from English to Portuguese independently by two professionals fluent in English. One was in the health profession and aware of the research objectives; the other had different healthcare training and was unaware of the research objectives. These were called the T1 and T2 translations.
- Step 2: Synthesis of translations: four authors (SVC, ISF, MDCL and VPR) integrated the translations (T1 and T2) and developed a single instrument called “synthesis T1/T2.”
- Step 3: Back-translation (B^1^T1/T2 and B^2^T1/T2): the synthesized instrument (T1/T2) was back-translated from Portuguese to English by two independent translators (not health professionals), who were double-blinded to the original version of the DTCQ-8 for translation purposes and to the concepts explored in the survey. Both were Brazilians and native Portuguese speakers who were proficient in English. They produced two versions of the back-translation (B^1^T1/T2 and B^2^T1/T2), which were compared with the original DTCQ-8. Dr. Nigel, an author of the original instrument and the DTCQ used in this research, approved the entire process of cross-cultural adaptation using best practices from another study [33, 34] to assess the various aspects of cross-cultural validity.
- Step 4: Committee of experts [35]: this committee, consisting of nine experts, was created to compare all the DTCQ-8 versions developed in the previous steps with the original DTCQ-8 questionnaire and produce a “pre-final” version of the DTCQ-8 in Brazilian Portuguese. This version was examined by the nine experts, who analyzed the synthesized version of the DTCQ-8; “T1/T2” was analyzed for face validity and content. Changes were made to the DTCQ-8 according to the suggestions of this committee. These changes resulted in a pre-final Portuguese version of the DTCQ-8. The versions, including the pre-final version in Portuguese and the back-translation, were returned to the experts and to the author of the original scale (Dr. Nigel) in a manner similar to that described in Table 2. All approved the pre-final version, which was the instrument used in step 5 (pre-test).
- Step 5: The pre-final version test (pre-test): the purpose of this step was to ensure that the adapted version retained content adequacy, writing clarity and usefulness in comparison to the original instrument, which was verified through research on its face validity and content for the target population. Although the instrument is self-administered, the researcher administered the pre-final version of the DTCQ-8 by reading it individually to the pre-final sample of 40 adult drug users. The researcher also utilized a form to record the users’ socio-demographics, clinical health and treatment status. In addition, the researchers used a specific instrument for semantic evaluation to record the understanding of each item and the meaning of the response provided by the target population [36]; this instrument evaluated the content’s validity for the target population.

Step 5 was administered in a room that reflected the dynamics of CAPSad. In this setting, the adapted DTCQ-8 version (Table 2) was ready for statistical evaluation of its psychometric properties, namely, reliability, face validity and content validity. The authors, who have conducted a clinical validation in the Brazilian context and are currently writing articles about the process, administered the adapted DTCQ-8 version of other drugs to a new sample—the final sample—and verified two psychometric properties: reliability (Cronbach’s alpha) and construct validity (contrasted groups and factor analysis).

### Analysis

#### Descriptive statistics

The descriptive statistics were obtained using absolute frequencies and percentages for three categorical variables and measures: average, standard deviation and median for numerical variables. The program used for typing and statistical calculations was the Statistical Package for Social Sciences (SPSS) version 21. In step 4, the expert committee obtained the satisfactory answers’ frequency on each of the items evaluated, and from the results of each item, the scores were averaged to provide blocks. To check the reliability of the adapted DTCQ-8 version for other drugs, the relative internal consistency measure, Cronbach’s alpha, was obtained for the pre-final sample. The data were double-entered by double-blinded typists.

#### Face validity of the DTCQ-8 as assessed by experts and by the target population of the pre-final sample

The face validity of the adapted DTCQ-8 was obtained through steps 4 and 5. We asked the users and experts who participated in this study the following question: “To Sir/Madam, does the instrument clearly measure (appear to measure) the trust a drug user has in his or her confidence to resist the urge to use drugs in high-risk situations?” All said yes.

#### Validity of DTCQ-8 content by experts and by the target population of the pre-final sample

Content validity was determined by experts through semantic, experiential, and conceptual evaluation, and the concordance index (CI) was calculated. A value greater than or equal to 0.80 is considered a parameter agreement among experts in this research [34]. Content validity by the target population was conducted by applying the semantic evaluation tool used in [36].

#### Reliability of DTCQ-8 from data collected with the pre-final sample

The instrument’s internal consistency and reliability were verified by Cronbach’s alpha; values greater than 0.70 were considered acceptable [37–41]. The Cronbach’s alpha was calculated using a total of eight items; the exclusion of each item was used to identify the clinical behavior of the data from these changes to identify possible weaknesses in the instrument’s internal consistency. In addition, we calculated the Spearman correlation coefficient to verify each item’s influence on the internal consistency of the total instrument.

## Results

### Participant demographics

The total sample (*n* = 40) comprised 20 Brazilian men and 20 women. The average age at the time of the study was 44.5 ± 9.32 years old. The substances used were crack and tobacco; these were responsible for their substance abuse problems and what motivated their treatment at the Psychosocial Care Center for Alcohol and Other Drugs (CAPSad). The participants’ characteristics are shown in Table 1.

**Table 1:** Socio-demographic characteristics of the 40 drug users in the pre-final sample

Variable	Group				Total group		*p* value
	Crack		Tobacco				
	N	%	N	%	N	%	
TOTAL	20	100	20	100	40	100	
Age: Average and SD (years)	38.35 ± 6.38		50.50 ± 7.26		44.43 ± 9.13		p^(1)^ < 0.001*
Age group							
Up to 39 years old	12	60	2	10	14	35	p^(2)^ < 0.001*
40–49	8	40	6	30	14	35	
50–65	-	-	12	60	12	30	
Gender							
Male	19	95	1	5	20	50	p^(2)^ < 0.001*
Female	1	5	19	95	20	50	
Color							
White	3	15	6	30	9	22.5	p^(3)^ < 0.323*
Black	3	15	5	25	8	20	
Brown	14	70	9	45	23	57.5	
Marital Status							
Single	11	55	11	55	22	55	p^(1)^ = 0.001
Married/Stable Union	9	45	9	45	18	45	
Schooling							
Illiterate	2	10	-	-	2	5	p^(3)^ = 0.021*
Up to 4 years	10	50	4	20	14	35	
5–8 years	5	25	5	25	10	25	
9 years or more	3	15	11	55	14	14	
Occupation							
Unemployed	6	30	7	35	13	32.5	p^(2)^ = 0.811
Officially registered	5	25	6	30	11	27.5	
Informal work	9	45	7	35	16	40	
Income							
No income	3	15	3	15	6	15	p^(3)^ = 0.772
Less than Brazil’s minimum wage	8	40	6	30	14	35	
Minimum wage of Brazil	4	20	7	35	11	27.5	
More than Brazil’s minimum wage	5	25	4	20	9	22.5	

Source: Original compilation. (*): Significant difference to the level of 5.0 %. (1): Student’s *t* test. (2): Chi-square Test of Pearson. (3): Fisher’s exact test

The age at first drug use among the crack users group was as follows: 15 % began under 11 years old, 35 % between 12 and 14 years old, 35 % between 15 and 17 and 15 % between 18 and 65. The average age at first drug use was 13.85 years old, and that of first crack use was 28.50 years old (SD: 7.30 years). Of the total group, 70 % began their consumption of psychoactive substances with alcohol, 20 % with marijuana and 10 % with tobacco. The drug with the second most common use was marijuana (45 %), followed by tobacco (30 %). Crack was the most popular drug with the third (43.7 %), the fourth (63.6 %), or fifth (33.3 %) most prevalent contacts. Aspirated cocaine was also used during the second (5 %), fourth (18.2 %) and fifth (66.7 %) contacts. Thus, all users began their consumption of psychoactive substances by using crack or snorting cocaine. In addition, 45 % reported they had been imprisoned in public jails, 55.55 % practiced urban violence, and 44.44 % engaged in domestic violence.

In the group of tobacco users, 20 % began at 11 years old, 60 % began between 12 and 13, 5 % between 14 and 17 and 15 % between 18 and 65. The average age of first use was 13.95 years old (SD: 3.99 years). Of these, 95 % started their consumption of psychoactive substances with tobacco and 5 % with alcohol. All 20 smokers used only these two substances. No tobacco users had been arrested or had issues with the police.

Of the crack users, 85 % had attended other treatment programs for their problem with this substance, 15.0 % were in their first treatment, 23.5 % had attended one or two treatments, 41.2 % had attended three or four treatments, and 35.3 % had attended more than 5 treatment programs. In addition, 41.2 % abandoned the treatment due to a crack use relapse, and 58.8 % abandoned the treatment because they considered it unnecessary. These individuals believed they were well or that the treatment was not providing improvements. None of the respondents were discharged from therapy.

Of the tobacco users, 40 % had participated in other treatments for smoking, 60 % were in their first treatment, 75 % had participated in one or two treatments, and 25 % had participated in three or four treatments. Of the total users, 75 % abandoned the treatment because they did not see results and did not want to stay in treatment or quit smoking.

### Assessment of face and content validity by the committee of experts

All of the experts reported that the adapted DTCQ-8 version for other drugs reliably measures drug users’ confidence to resist the urge to consume these substances in high-risk situations. The assessment of the experts regarding the equivalence is shown in Table 2.

**Table 2:** Evaluation of the equivalences of the pre-final version of DTCQ-8 for other drugs

	Semantic equivalence					
Item						
	1.1^a^	1.2^a^	1.3^a^	Idiomatic Equivalence	Experiential Equivalence	Conceptual Equivalence
A1^b^	1.000	1.000	1.000	0.778	0.889	0.889
A2^b^	1.000	1.000	1.000	1.000	0.889	1.000
1	1.000	1.000	1.000	1.000	1.000	1.000
2	1.000	1.000	1.000	1.000	0.889	1.000
3	1.000	1.000	1.000	1.000	1.000	1.000
4	0.889	0.889	1.000	0.889	0.889	0.889
5	1.000	1.000	1.000	1.000	1.000	1.000
6	1.000	1.000	1.000	1.000	1.000	1.000
7	1.000	1.000	1.000	1.000	1.000	1.000
8	1.000	1.000	1.000	1.000	1.000	1.000
Average of items	0.989	0.989	1.000	0.967	0.956	0.978

^a^1.1 correct writing, 1.2 equivalent significance, 1.3 grammatically correct

^b^Explanatory paragraphs on the scale and filled

The concordance index for idiomatic equivalence of the A1 item was 0.778 due to a specialist’s modification suggestion that was not accepted (A1 item: “Listed below are situations in which some people experience problems with drug use”).

The evaluation by this study’s specialists led to modifications of the A1, 1, 4, 5, 6 and 7 items, which were adjusted in accordance with the instructions received (Table 3); this resulted in the pre-final Portuguese version of the DTCQ-8 for additional drugs (Table 4).

**Table 3:** Modified items from the suggestions of the Committee of experts

Original DTCQ-8	Itens da Versão “Síntese T12” DTCQ-8	Itens da Versão pré-final DTCQ-8
A1. Imagine yourself as if you are right now in each of these situations.	A1. Imagine-se você agora mesmo em cada uma dessas situações.	A1. Imagine-se agora em cada uma dessas situações.
1. If I were angry at the way things had turned out.	1. Se eu estivesse com raiva pelo rumo que as coisas tomaram.	1. Se, por algum motivo, eu estivesse com raiva.
4. If I wanted to find out whether I could use _________ occasionally without getting hooked.	4. Se eu quisesse me testar se consigo usar _____ às vezes sem me viciar.	4. Se eu quisesse testar se consigo usar __________, às vezes, sem me tornar dependente.
5. If I unexpectedly found some __________ or happened to see something that reminded me of __________.	5. Se eu por acaso encontrasse alguma droga __________ ou visse alguma coisa que me lembrasse a droga _________.	5. Se eu, por acaso, encontrasse alguma droga __________ ou visse alguma coisa que me lembrasse o ato de usar _________.
6. If other people treated me unfairly or interfered with my plans.	6. Se outra pessoa me tratasse mal ou atrapalhasse meus planos.	6. Se alguém me tratasse injustamente ou atrapalhasse meus planos.
7. If I were out with friends and they kept suggesting we go somewhere and use _________.	7. Se eu estivesse com amigos e eles insistissem para sairmos para usar ____________.	7. Se eu estivesse com amigos e eles insistissem para irmos a algum lugar para usar ____________.

**Table 4:** Comparison of the original version of the DTCQ-8 and cross-cultural adaptation

Original DTCQ-8	Versão pré-final em português DTCQ-8	Retrotradução DTCQ-8
1. If I were angry at the way things had turned out.	1. Se, por algum motivo, eu estivesse com raiva.	1. If, for some reason, I was angry.
2. If I had trouble sleeping.	2. Se eu tivesse problemas para dormir.	2. If I had trouble sleeping.
3. If I remembered something good that had happened.	3. Se eu lembrasse de alguma coisa boa que tivesse acontecido.	3. If I remembered something good that had happened.
4. If I wanted to find out whether I could use _________ occasionally without getting hooked.	4. Se eu quisesse testar se consigo usar __________, às vezes, sem me tornar dependente.	4. If I wanted to test if I could use _______, sometimes, without becoming dependent.
5. If I unexpectedly found some __________ or happened to see something that reminded me of __________.	5. Se eu, por acaso, encontrasse alguma droga __________ ou visse alguma coisa que me lembrasse o ato de usar _________.	5. If I, by chance, found some drug _______ or saw something that reminded me of the act of using _________.
6. If other people treated me unfairly or interfered with my plans.	6. Se alguém me tratasse injustamente ou atrapalhasse meus planos.	6. If somebody treated me unfairly or interfered with my plans.
7. If I were out with friends and they kept suggesting we go somewhere and use _________.	7. Se eu estivesse com amigos e eles insistissem para irmos a algum lugar para usar____________.	7. If I was with some friends and they insisted to go some place to use __________.
8. If I wanted to celebrate with a friend.	8. Se eu quisesse comemorar com um amigo.	8. If I wanted to celebrate with a friend.

The evaluation based on clarity of the language (0.972), practical relevance (0.958) and theoretical relevance (0.958) showed average satisfactory scores (Table 5).

**Table 5:** Content validity of the pre-final version of the DTCQ-8 for other drugs

Item of DTCQ-8	Clarity of language	Practical relevance	Theoretical relevance
1	1.000	0.889	0.889
2	0.778	0.889	0.889
3	1.000	0.889	0.889
4	1.000	1.000	1.000
5	1.000	1.000	1.000
6	1.000	1.000	1.000
7	1.000	1.000	1.000
8	1.000	1.000	1.000
Average of the items	0.972	0.958	0.958

### Face and content validity (semantic evaluation) for the target population

All users reported that the adapted DTCQ-8 version for other drugs measured their confidence to resist the urge to use drugs in high-risk situations. All users understood that item 2 was referring to not being able to sleep. Most participants asked the question: “Do I have confidence to say that I will not use?” In Section 4, most participants asked: “What if I wanted to test myself?”

On items 7 and 8, some users said they did not accept invitations for drug use and did not celebrate with friends, requiring the researcher’s intervention regarding the scale filling instructions. DTCQ-8 assumes the mental anticipation of high-risk situations, i.e., the user needs to imagine the situation at hand.

Most users needed clarification of items 4, 7 and 8. They claimed they did not test themselves and did not accept invitations because they knew they would not resist the urge to use drugs in these situations.

### DTCQ-8 version for other drugs reliability 

The results of the DTCQ-8 version of the reliability for other drugs were obtained from Cronbach’s alpha; these are described in Table 6.

**Table 6:** Cronbach’s alpha values and Spearman’s correlation coefficient of the DTCQ-8 for other drugs

DTCQ-8 for other drugs	Total item correlation^a^ r	Alpha if item deleted
1. If, for some reason, I was angry	0.780	0.837
2. If I had trouble sleeping	0.871	0.863
3. If I remember anything good that happened	0.675	0.818
4. If I wanted to test whether I can use ______ sometimes without becoming dependent on it	0.883	0.869
5. If I find any drugs of _______ or see something that I could remember the act of using _______	0.551	0.815
6. If someone treats me unjustly or gets in the way of my plans	0.890	0.868
7. If I was with friends and they insisted on going somewhere for use of _______	0.665	0.786
8. If I wanted to celebrate with a friend	0.877	0.876

Source: Own compilation. ^a^Spearman’s rank correlation coefficient. Note: Cronbach’s Alpha for the complete instrument was 0.889

Given the above observations, the pre-final Portuguese version of the DTCQ-8 for other drugs, its back-translation and the comparison with the original showed a satisfactory translation of the instrument under consideration because the differences were derived from the cross-cultural adaptation to Brazil (Tables 3 and 4).

### Self-efficacy scores using the adapted DTCQ-8 version for other drugs

The average total scores of the self-efficacy to resist the urge to use drugs was 67.50 for crack users and 52.50 for tobacco users. Thus, 5 % of users were classified as having low self-efficacy, 67.5 % as moderate self-efficacy and 27.5 % as high self-efficacy (Table 7).

**Table 7:** Self-efficacy scores according to the group that responded to the DTCQ-8

Items of DTCQ-8	Crack	Tobacco	*p* value
	Median (IQR)	Median (IQR)	
1	60 (75)	40 (75)	*p* ^(1)^ = 0.366
2	100 (15)	90 (60)	*p* ^(1)^ = 0.047*
3	100 (20)	90 (60)	*p* ^(1)^ = 0.201
4	20 (90)	20 (85)	*p* ^(1)^ = 0.987
5	80 (80)	40 (90)	*p* ^(1)^ = 0.134
6	80 (80)	40 (100)	*p* ^(1)^ = 0.380
7	70 (80)	70 (80)	*p* ^(1)^ = 0.911
8	90 (80)	100 (90)	*p* ^(1)^ = 0.956
DTCQ-8 complete	67.50 (46.87)	52.50 (58.13)	*p* ^(1)^ = 0.272

Source: Own compilation. (*): Significant difference to the level of 5.0 %. (1): Mann–Whitney test

We found that item 4 (“If I wanted to test if I can use ______ sometimes without becoming dependent”), which corresponds to “Domain: Personal control test,” had a lower average (35.50 + 40.46- DP) of self-efficacy to resist the urge to use drugs in this particular situation. In contrast, the highest average (78.50 ± 32.78-SD) of self-efficacy was in response to item 3 (“If I remember something good that happened”), which corresponds to “Domain: pleasant emotions,” demonstrating that item 3 does not represent a high risk for other drug users participating in the study.

The results in Table 8 demonstrate that it is possible to verify that the majority in each group had mild self-efficacy, with percentages ranging from 70 % for crack users to 65 % for tobacco users, followed by high self-efficacy, with percentages between 30 % and 25 %, respectively. However, there was no significant difference (*p* > 0.05) between groups regarding the participants’ self-efficacy level classification.

**Table 8:** Classification of the self-efficacy according to the group that answered the DTCQ-8

Classification of self-efficacy according to the average score of the DTCQ-8	Group				Group		*p* value
	Crack		Tobacco		Total		
	N	%	N	%	N	%	
Low (Average of the instrument < 20 %)	-	-	2	10	2	5	*p* ^(1)^ = 0.565
Moderate (Average of the instrument from 20 % to 80 %)	14	70	13	65	27	67.5	
High (Average of the instrument > 80 %)	6	30	5	25	11	27.5	
TOTAL	20	100	20	100	80	100	

Source: Own compilation. (1): Fisher’s exact test

## Discussion

### Participants’ demographics

The sociodemographic characteristics of this study were similar to those in other studies of cross-cultural adaptation of instruments for drug users [42, 43]. However, some methodological studies of cross-cultural adaptation and translation do not report the sociodemographic of their targeted population and focus solely on the gender [44] and other aspects of the study [45]. These studies demonstrate that the emphasis on methodological studies is the same as that of the method used in cross-cultural adaptations [46–48], which, in the present study, was the guideline by Beaton et al. (2007) [30]. Differences were identified concerning the sociodemographic characteristics among the targeted population of this study and the original DTCQ-8 article, education, type of consumed psychoactive substances and employment status.

The results of the current study results indicate that the average age of first cigarette consumption was lower than previously found in Brazilian research studies [6]. In addition, the beginning of crack use [49] was higher than in other Brazilian studies. There are similar results regarding the urban and domestic violence among crack users [6]. Urban violence is also associated with illicit drug trafficking and as a way of obtaining the substance [50].

### Assessment of face and content validity by the committee of experts

The translation process and cultural adaptation of measuring instruments take into account the target population’s context or culture to preserve the meaning of the construct assessed [51, 52]. An committee comprising professionals from various backgrounds contributed to the analysis of the instrument from different perceptions and perspectives [53, 54]. The changes made to the adapted instrument are considered reasonable and necessary because they facilitate understanding by the targeted population [55–58].

This process has shown that the instrument is easy to administer and can be completed in a satisfactory amount of time.

### Face and content validity for the targeted population

The face validity was similar to that reported in another Brazilian article [59]. There are studies that have the same targeted population and discuss the instrument regarding the aspects of face validity and content [36]. However, our article used the content validity method, as have other Brazilian studies [37–39].

### Confirmability of the adapted DTCQ-8 version for other drugs

Cronbach’s alpha yielded values similar to those of the original scale [26, 28], showing reliability of the instrument as applied to the Brazilian population in this study [37–39].

### Self-efficacy scores DTCQ-8 version for other drugs

The personal control test was identified as a high-risk and low-efficacy situation for resisting the desire to consume drugs, which is a result similar to that of other studies [60, 61]. Thus, when the users understand their behavior pattern and problems associated with the consumption of psychoactive substances, their self-efficacy increases and they become able to resist the urge of consuming drugs. The domain “pleasant emotions” was not considered a high-risk situation for consuming drugs because the users attained higher self-efficacy levels to resist the desire of using these drugs [62].

Therefore, stimulating the seeking of healthy habits that provide new forms of pleasure is an important strategy for behavioral change [63, 64].

Drug users’ behavioral dynamics on their confidence to resist the urge to consume these substances in high-risk situations was similar to those of other studies [65, 66].

The classification of users of other drugs in this study who have moderate self-efficacy was similar to that in other studies [67]; it is believed that this classification can be related to the behavioral dynamics of users seeking treatment [68].

## Conclusion

The adapted DTCQ-8 version for other drugs was translated, culturally adapted and validated for the pre-final sample; this is a useful tool for clinicians and researchers in the evaluation of drug user self-efficacy to resist the urge to consume these substances in high risk situations. Furthermore, this study contributes to the planning of interventions, the monitoring of changes in users and the evaluation of treatment effectiveness.

The present research is limited by presenting the reliability and validity evaluation as an adapted DTCQ-8 version for other drugs in which the psychometric properties have only been tested for pre-final sample.

This limitation will be rectified in our next article regarding the clinical validation of the adapted DTCQ-8 version for other drugs for use in Brazil. The clinic validation was made with a new sample of drug users in CAPSad. The content’s validity was evaluated through contrasted groups and factor analysis, and the instrument’s internal consistency was verified by Cronbach’s alpha.

### Ethics approval and consent to participate

The study was approved by the Ethics Committee in Research with Human Beings–UFPE under CAAE 31677114.0.0000.5208. We received permission to use this instrument from the Centre for Addiction & Mental Health (CAHM) and obtained written informed consent from all participants (drug users and experts).

### Consent to publish

All authors read and approved the final version of the manuscript.

All authors have consented to publication of this manuscript.

All study participants signed free and informed consent. They also agreed to publish the data.

### Availability of data and materials

If you want access to complete data on the doctoral thesis, contact the corresponding author by email selumares@gmail.com.

## Abbreviations

DTCQ-8: Drug-taking confidence questionnaire
CAPSad: Day-care center for alcohol and other drugs
